# Reducing Reading Anxiety and Enhancing Reading Attitudes Through Interactive Read-Aloud: An Action Research Study with Second-Grade Students

**DOI:** 10.3390/bs16071138

**Published:** 2026-07-07

**Authors:** Mustafa Ozturk, Muhammet Sonmez, Mucahit Durmaz, Abdurrahman Baki Topcam, Fatih Cetinkaya, Timothy Rasinski

**Affiliations:** 1 Grand National Assembly of Türkiye, Ministry of Education, Ankara 06530, Türkiye; mustafa.ozturk2@tbmm.gov.tr; 2 Hacı Seyit Taşan Primary School, Ministry of Education, Kocaeli 41455, Türkiye; muhammetsnmz41@gmail.com; 3 Nurettin Topçu Training and Practice Center, National Education Academy, Ministry of Education, Ankara 06170, Türkiye; 4 Şanlıurfa Science and Art Center, Ministry of Education, Sanliurfa 63200, Türkiye; bakitopcam96@gmail.com; 5 Faculty of Education, Duzce University, Duzce 81620, Türkiye; cetincetinkaya@duzce.edu.tr; 6 Department of Teaching, Leadership, and Curriculum Studies, College of Education and Human Ecology, Kent State University, Kent, OH 44242, USA; trasinsk@kent.edu

**Keywords:** reading, anxiety, reading attitude, interactive reading

## Abstract

The study recommended implementing teacher-led, face-to-face interactive read-aloud activities with children’s picture books to support students with high reading anxiety and negative attitudes toward reading. The study employed an action research design and included a sample of 32 second-grade primary school students. The students participated in a 13-week interactive read-aloud program designed to address reading anxiety and reading attitudes. Picture storybooks were used as instructional materials throughout the intervention. The students’ reading anxiety was measured with the Reading Anxiety Scale, while their reading attitudes were assessed using the “Garfield” Visual Reading Attitude Scale for Grades 1–6. In addition, process-oriented qualitative data were obtained through a researcher’s journal. Statistical analyses revealed that (1) second-grade students’ reading anxiety scores decreased significantly after participation in the interactive reading program, (2) their reading attitude scores became significantly more positive, and (3) the qualitative findings indicated that the students developed positive experiential responses during the interactive reading process.

## 1. Introduction

Reading is a fundamental language skill that involves not only cognitive processes but also affective elements. Readers must manage the complex interplay of semantic, phonological, syntactic, and strategic elements as they process various dimensions of text to achieve different goals during reading (Cartwright, 2009). Furthermore, affective and motivational factors also influence reading comprehension, making the process more complex (Cartwright et al., 2015).

Although cognitive skills form the foundation of reading, they do not fully account for students’ reading proficiency or the underlying causes of reading difficulties (Boakye et al., 2014). Bernhardt (2005) argues that approximately 50% of reading proficiency is attributable to affective issues, while the other 50% is linked to vocabulary knowledge and grammatical structures. In addition to cognitive and language-based factors, non-cognitive factors, including affective variables such as self-regulation, attitude, self-efficacy, and anxiety, can also explain differences in reading performance (Carroll & Fox, 2016; Denton et al., 2020; Toste et al., 2020). Affective factors support the multidimensional structure of reading proficiency and offer important clues about the causes of reading difficulties students encounter (Boakye et al., 2014). While students who develop a positive attitude toward reading and experience low anxiety are expected to show more positive reading-related outcomes, it has been noted that negative situations can lead to disengagement from the reading process (Bakkaloğlu, 2023). In light of this evidence, the present study focused on readers’ anxiety levels and attitudes toward reading.

The present study is also informed by Duke and Cartwright’s (2021) active view of reading, which extends the simple view of reading by emphasizing that reading is not limited to word recognition and language comprehension but also involves active self-regulatory processes, including motivation, engagement, executive functioning, and strategy use. From this perspective, affective variables such as reading anxiety and reading attitudes are not peripheral to reading development; rather, they are closely related to how students participate in, regulate, and experience reading.

While it is difficult to completely eliminate reading anxiety, mild levels of anxiety may, in some cases, serve to motivate students. However, the degree of this anxiety may vary among individuals. D. A. Francis et al. (2019) define reading anxiety as intense fear or apprehension that occurs in situations requiring the processing of written information; they emphasize that this anxiety causes students to develop reluctance toward reading tasks and, as a result, their reading performance may be negatively affected. Similarly, Edwards et al. (2023) argue that high levels of reading anxiety result in lower levels of reading performance. They claim that negative evaluations of students’ reading abilities trigger feelings of inadequacy and apprehension, which, in turn, inhibit existing cognitive capacities. Reading anxiety is thus considered to have a bidirectional nature: while it may contribute to poor reading performance, low reading skills may also give rise to anxiety (Piccolo et al., 2017; Ramirez et al., 2019).

It is known that students who develop a positive attitude toward reading engage in reading more frequently and are more willing to participate in reading activities. Furthermore, research has shown that these students demonstrate higher reading comprehension levels compared to their peers (Kush et al., 2005; Kuşdemir & Katrancı, 2016). Therefore, it is necessary to develop practices to reduce students’ reading anxiety and support positive reading attitudes.

Lane and Wright (2007) emphasize that social and emotional literacy interactions may foster positive attitudes toward reading and motivate children to participate in literacy activities independently. In the present study, interactive read-aloud practices refer to teacher-led, face-to-face reading sessions in which children’s picture books are read aloud and students actively participate through predictions, questions, personal connections, and classroom discussions (Ceyhan & Yıldız, 2021; Fox, 2008; Ivey, 2003; Trelease, 2013). Therefore, in the present study, interactive literacy practices are considered as a potential solution to the problems related to the phenomenon of reading anxiety and to the affective components of reading attitudes.

### 1.1. Theoretical Framework

The theoretical framework of the present study is grounded in the affective and sociocultural dimensions of reading. Reading anxiety is conceptualized as students’ fear, apprehension, or discomfort in reading-related situations, whereas reading attitude refers to students’ interest, enjoyment, and willingness to engage in reading. From a sociocultural perspective, these affective dimensions are shaped not only by individual experiences but also through classroom interaction, teacher guidance, peer participation, and meaningful literacy practices. Therefore, interactive read-alouds are considered in this study as socially mediated instructional contexts that may reduce students’ reading anxiety and support more positive reading attitudes.

Within this theoretical framing, the intervention was organized around interactive read-aloud practices, which had been shown to support various student skills. This approach aims to encourage both reader and listener to reflect on the text and ensure active participation of both parties in the process (Çetinkaya et al., 2023). An interactive read-aloud is defined by Meller et al. (2009) as the conscious and prosodic reading of children’s books aloud by a practitioner such as a teacher or parent. According to Ceyhan and Yıldız (2021), in this process, the teacher provides think-aloud examples before, during, and after reading and models comprehension strategies by demonstrating how to make predictions, ask questions, identify the main idea, establish connections with the text, and monitor understanding. Furthermore, under the teacher’s guidance, students make predictions about the text, establish semantic relationships, pose questions, identify the main idea, check their predictions, and engage in critical/evaluative thinking.

Students are expected to participate actively in interactive read-alouds, as such engagement helps them feel included in the community, access content they might not be able to reach independently, and enjoy the shared reading experience (Alatalo et al., 2024; Çetinkaya et al., 2023; Venegas & Guanzon, 2023). Interactive read-aloud practices require reciprocal communication, thereby motivating students to participate and fostering a socially rich classroom environment (Öksüz et al., 2025). Since meaning in interactive read-aloud is constructed through interaction—in other words, through socialization—this practice also aligns with Vygotsky’s sociocultural theory of development (Çetinkaya et al., 2023).

Accordingly, this study primarily adopts a sociocognitive stance toward reading affect, as reading anxiety and reading attitudes are examined as measurable affective constructs associated with students’ reading experiences. At the same time, because interactive read-alouds are socially mediated classroom practices, the study also acknowledges the situated nature of students’ motivation, engagement, and affective responses during reading. A situated perspective suggests that students’ motivation and affect are shaped through participation in social contexts rather than being located only within the individual. Nolen et al. (2015) argue that motivation should be examined in relation to context, identity, meanings, and social participation. This perspective is particularly relevant to interactive read-aloud practices, as students’ affective responses to reading may develop through shared discussion, teacher guidance, peer interaction, and collective meaning-making.

Interactive reading practices initiated and guided by a teacher provide facilitating elements for students whose reading anxiety is outside the optimal range. The presence of a leader managing the process enables students to analyze texts collectively or with guidance rather than on their own, thereby reducing their fear of making mistakes (Wasik & Bond, 2001). In interactive reading, participation occurs not only through reading aloud but also through predictions, interpreting illustrations, noting interesting vocabulary, and responding to questions (Ceyhan & Yıldız, 2021). By creating supportive environments, the negative impact of challenges such as reading anxiety can thus be minimized (Katzir & Shany, 2010).

### 1.2. Literature Review

Reading is a complex process that involves textual elements, contextual conditions, environmental factors, and reader characteristics. Reader characteristics include automaticity and fluency, prior knowledge, vocabulary, motivation, positive self-perception, and attitudes toward reading (Fletcher et al., 2021; Ghaith, 2017; Peng et al., 2018; Seidenberg, 2017). Underdevelopment of reading proficiency, including accurate reading, fluent reading, text comprehension, language comprehension, and motivation, have been attributed to various causes (Barth & Elleman, 2017; Hoover & Tunmer, 2018). Two of these reasons are particularly linked to emotional well-being: anxiety (Fletcher & Grigorenko, 2017) and students’ attitudes toward reading. Indeed, a recent meta-analysis demonstrated a statistically significant and moderate relationship between poor reading and reading anxiety (D. A. Francis et al., 2019).

Reading attitude refers to students’ interest, enjoyment, and willingness to engage in reading. Students with positive reading attitudes tend to read more frequently and participate more willingly in reading activities, and reading attitude has been associated with reading achievement, reading amount, reading speed, and comprehension (Andini & Yukamana, 2024; Kush et al., 2005; Kuşdemir & Katrancı, 2016; Soysal, 2022; Xiao, 2023). However, reading attitudes may also vary depending on students’ classroom experiences, access to meaningful texts, and opportunities for social interaction around reading. In recent years, the number of studies on reading anxiety and reading attitudes have been increasing. Fishstrom et al. (2024) revealed that reading anxiety is significantly correlated with both general anxiety and test anxiety, and that high reading anxiety negatively affects word reading fluency, text fluency, and comprehension skills. Andini and Yukamana (2024) stated that there is a moderate relationship between students’ reading attitudes and reading achievement. Xiao (2023) emphasized that reading attitude shows significant relationships with both reading self-perception and reading achievement, and that the relationship between attitude and achievement is mediated by the amount of reading. Soysal (2022) showed that there are strong and positive relationships between students’ reading speed and reading attitudes, as well as between comprehension levels and reading attitudes. Macdonald et al. (2021) demonstrated that reading accuracy, fluency, and comprehension skills are significantly negatively correlated with reading anxiety. Trisnayanti et al. (2020) found that when students experience high reading anxiety, their reading self-efficacy and reading proficiency are low; they also observed that both anxiety and self-efficacy, when considered together, were significant predictors of reading proficiency. Ramirez et al. (2019) found that the relationship between reading anxiety and achievement differed by gender, with male students being more susceptible to the negative effects of reading anxiety on achievement. They also reported that reading achievement was more strongly related to reading anxiety than to positive reading experiences, which may be due to the greater psychological impact of negative experiences.

Beyond correlational studies examining the relationships among reading anxiety, reading attitudes, and reading-related outcomes, intervention-based research has also investigated whether targeted programs can reduce reading anxiety or support reading-related affect. D. Francis et al. (2021) demonstrated that a 12-week PRAX intervention (for both low reading skills and anxiety) increased children’s reading and spelling accuracy and simultaneously reduced anxiety disorders and their symptoms. Similarly, Valizadeh (2021) demonstrated that an 11-week reading program based on English reading comprehension strategies was effective in reducing reading anxiety in EFL students. However, research on reading anxiety and attitudes appears to be largely correlational. Therefore, the need for applied interventions aimed at reducing students’ reading anxiety and fostering positive reading attitudes is clear. Interactive read-alouds offer a supportive classroom context in which students listen to texts, respond to questions, make predictions, share personal connections, and construct meaning with teachers and peers. Previous studies show that interactive read-alouds support reading comprehension, fluency, vocabulary, motivation, participation, and social interaction in literacy activities (Ceyhan & Yıldız, 2021; Çetinkaya et al., 2018; Lane & Wright, 2007; McClure & Fullerton, 2017; Wiseman, 2011).

Therefore, interactive read-aloud activities not only contribute to the development of skills and literacy but also allow students to develop, express, and defend their ideas within the classroom (Wiseman, 2011). Greene Brabham and Lynch-Brown (2002) emphasized that the success of interactive read-alouds in language and literacy development relies on the social interactions between the teacher and students and the feedback given during this process. The conversations that take place during this process not only guide participation in reading activities but also comprise the fundamental elements of reading instruction (Justice et al., 2009; Santoro et al., 2008). Students who participate in interactive read-alouds make progress in their comprehension of the text and their attitudes toward learning (Greene Brabham & Lynch-Brown, 2002). McClure and Fullerton (2017) stated that interactive read-alouds provide students with the opportunity to verbally share their thoughts and understanding and provide them with the opportunity to use their social energy to listen to the opinions of others. Accordingly, the social environment created by interactive read-alouds can reduce students’ anxiety by making them feel comfortable and strengthening their positive attitudes toward reading. The social context created in such practices supports students’ more effective interactions with their peers, teachers, and books.

Interactive read-aloud activities provide a supportive environment where students can experiment with comprehension strategies they can use in their independent reading and construct meaning together (Maloch & Beutel, 2010). In this environment, the guidance of a teacher or parent who manages the read-aloud process provides special opportunities to make reading enjoyable for students who perceive their reading skills as inadequate. Thus, students experiencing high anxiety due to low reading proficiency can move beyond their anxiety and focus on the aesthetic dimensions of reading. As emphasized by Ceyhan and Yıldız (2021), students are more engaged in the reading process when they believe they can achieve success, collaborate with their peers, feel they are competent readers, and have opportunities to make choices. The interaction, shared meaning-building, and collaborative opportunities offered by interactive read-alouds reduce students’ anxiety about making mistakes and enable them to actively participate in the process. Therefore, interactive read-alouds are appropriate for this study because they may reduce the pressure of individual reading and support students’ reading-related affect.

### 1.3. The Present Study

Studies have revealed that interactive reading activities are effective in developing basic language skills such as reading, writing, speaking, and listening (Çetinkaya et al., 2018; Martin-Chang & Gould, 2012; Sönmez et al., 2023), mathematics (McGuire et al., 2021), STEM education (Ciecierski et al., 2017), science (Durmaz et al., 2023), and the arts (Wong et al., 2021). In addition to their academic contributions, interactive read-aloud practices are also known to make a significant contribution to students’ acquisition of positive behaviors and support their emotional well-being by providing social-emotional learning opportunities (Britt et al., 2016). Based on this theoretical positioning, interactive read-aloud practices were considered not only as instructional activities but also as socially mediated experiences through which students’ reading-related affect could be supported. Accordingly, the aim of the present study was to develop primary school students’ affective skills by reducing anxiety and improving their attitudes towards reading through interactive read-aloud activities. For this purpose, the following research questions were addressed:

1. How do interactive read-aloud practices affect elementary school students’ reading anxiety levels?
2. How do interactive read-aloud practices affect elementary school students’ attitudes toward reading?

## 2. Materials and Methods

This study was designed as an action research project using both quantitative and qualitative data. Quantitative data were collected through pre-test and post-test measures of reading anxiety and reading attitudes, while qualitative data were obtained from the teacher’s research journal to describe students’ responses during the intervention process.

### 2.1. Research Design

The study was designed and conducted as an action research project. The choice of action research was guided by a practice-based problem identified by the classroom teacher: students read infrequently, were hesitant to read aloud in group work, had limited access to children’s books at home, and showed high reading anxiety and negative attitudes toward reading. Action research is considered a practice-based research model, particularly suitable in contexts where researchers are actively involved in the problem situation (Patton, 2002). This study employed a technical-scientific-collaborative action research design. The primary aim of this approach is to test or evaluate an application within a pre-determined theoretical framework. In the present study, this framework refers to the affective and sociocultural dimensions of reading, particularly the ways in which reading anxiety and reading attitudes may be shaped through socially mediated literacy practices. Accordingly, this design enabled the researchers to evaluate the interactive read-aloud intervention within the predetermined theoretical framework while also documenting process-oriented observations during implementation. Under the guidance of a researcher familiar with the theoretical framework, the practitioner can implement a new approach, and this process can be analyzed by the researcher to provide an evaluation of the application. There is close interaction between the practitioner and the researcher during the application process. Problems encountered in the application are communicated to the researcher, and the researcher conveys solutions within their area of expertise to the practitioner. The practitioner continues the application in line with the suggestions (Yıldırım & Şimşek, 2016).

### 2.2. Participant

The study sample consisted of 32 second-grade students. Nineteen of the participants were boys and thirteen were girls, and the students were between the ages of 7 and 8 and attending a public school in Turkey.

#### Data Collection

Ethical approval for this study was obtained from an independent university ethics committee prior to data collection. Participation in the study was voluntary, and informed consent was obtained from the parents or legal guardians of all participants. All procedures were conducted in accordance with ethical standards for research involving human participants.

Data for the study were collected using the Reading Anxiety Scale developed by Çeliktürk and Yamaç (2015) and the “Garfield” Visual Reading Attitude Scale for Grades 1–6, adapted into Turkish by Kocaarslan (2016). Both instruments are previously developed and validated scales rather than single-item questions. The Reading Anxiety Scale was used to assess students’ anxiety, fear, and discomfort in reading-related situations, whereas the “Garfield” Visual Reading Attitude Scale was used to assess students’ affective orientation toward reading through child-friendly visual response options. Because the participants were second-grade students, the scales were administered with appropriate classroom guidance to ensure that students understood the instructions and response format. In addition, to capture process-oriented observations during the interactive reading sessions, the classroom teacher maintained a research journal documenting the implementation. These instruments were selected because they directly corresponded to the two affective constructs examined in the study: reading anxiety and attitudes toward reading.

In response to this practice-based problem, a 13-week intervention process was planned and implemented. The books used during the interactive reading sessions were selected from children’s literature focusing on books and reading. During the selection process, students’ age and grade levels were taken into account. Selected books were reviewed by experts in children’s literature and incorporated into the intervention based on their suitability. For example, one of the books used in the process, *Madeline Finn and the Library Dog* portrays a child struggling with reading and lacking self-confidence who improves their reading skills with the help of a non-judgmental listener, the library dog. The chosen books were designed to target the affective dimensions of reading for the participants.

Throughout the 13-week period, one book designated by the classroom teacher was read each week. The plans were prepared according to the framework presented by Rasinski and Smith (2018) for interactive read-aloud plans. Interactive reading plans were developed by the researchers and structured into two sessions of 40 min each. The plans included pre-reading activities (such as activating background knowledge and introducing key vocabulary), interactive questioning during reading, and post-reading discussions. To make the read-aloud an interactive experience, the teacher used prediction questions, think-aloud strategies, and encouraged students to share personal connections with the story. Students were also invited to ask questions and participate in short discussions throughout the reading. All instructional materials included in the plans were prepared by the researchers and provided to the classroom teacher. Bibliographic information about the books used in the process is presented in Table 1. The books were used in their Turkish editions during the implementation of the intervention process; their English titles are provided in the table for international readability.

### 2.3. Data Analysis

The quantitative data obtained in the study were subjected to normality tests to determine which statistical analyses to apply. According to Tabachnick and Fidell (2019), for the data to be considered normally distributed, the skewness and kurtosis values must lie between −1.5 and +1.5.

As shown in Table 2, the data demonstrated a normal distribution. Accordingly, the quantitative data were analyzed using the dependent samples *t*-test, whereas the qualitative data obtained from the teacher’s journal were examined through content analysis.

The qualitative data obtained from the teacher journal were analyzed through content analysis. The teacher wrote their journals in Turkish during the implementation process. However, the quotations included in the findings have been translated from Turkish into English. In this process, the journal entries recorded during the interactive reading activities were reviewed in detail, and students’ responses were identified and coded according to similar expressions and observations. To ensure the reliability of the qualitative data obtained during the research process, the coding reliability was tested. Accordingly, the data were coded by a field expert. By comparing the coding done by the researcher and the field expert, codes with agreement and disagreement were identified. Coding reliability was calculated according to the formula proposed by Miles and Huberman (1994), and the resulting reliability rate was determined as [Agreement/(Agreement + Disagreement)] 84% [(49/49 + 12)]. The resulting codes were then grouped under broader response categories based on their shared characteristics, and the frequency of each category was calculated. This analysis made it possible to describe recurring patterns in students’ reactions during the intervention process, particularly their emotional responses, personal connections, willingness to participate, and other observable forms of engagement. The qualitative findings were presented with direct excerpts from the journal in order to support and illustrate the identified categories. For example, students’ statements such as wanting to touch a book, applauding after a reading, or expressing excitement during the activity were coded as emotional reactions. Requests such as “Can I read the next page?” were coded as desire to participate, while references to previously read books were coded as intertextual connections. 

## 3. Findings

Table 3 and Table 4 show the dependent samples *t*-test findings for comparing the participants’ reading attitude and reading anxiety pre-test and post-test scores.

Table 3 presents the results of the dependent samples *t*-test comparing the participants’ pre-test and post-test reading attitude scores. According to the findings, the participants’ average pre-test score was 3.13, while this value increased to 3.46 in the post-test. Considering the significance level, there was a statistically significant difference between the pre-test and post-test scores (t(31) = −2.98, *p* < 0.05).

Table 4 shows the dependent samples *t*-test results for comparing the participants’ reading anxiety pre-test and post-test scores. According to the findings, the mean reading anxiety score decreased from 2.53 in the pre-test to 2.04 in the post-test. Considering the significance level, there was a statistically significant difference between the participants’ reading anxiety scores in favor of the pre-test and post-test (t(31) = −2.98, *p* < 0.05).

### Observations Regarding the Intervention Process

Table 5 presents students’ responses reflected in the teacher’s journal during the book reading process.

The qualitative findings were interpreted in relation to the quantitative results. Students’ requests to read the next page, touch the books, and participate in discussions were consistent with the increase in reading attitude scores. Similarly, students’ applause, excitement, and willingness to engage with books suggested that the interactive read-aloud process provided a less threatening reading context, which was consistent with the decrease in reading anxiety scores.

Based on the findings recorded in the teacher’s journal, the students primarily exhibited emotional reactions during the interactive reading of the books. They related events in the books to their own experiences, showed willingness to participate in the reading activity, empathized with the protagonists, were influenced by the structural features of the books, and, in one observed instance, made an intertextual connection.

For example, one entry from the journal states:

“When I brought the book *Library Lion* to the classroom, the students wanted to touch it. While showing the pictures, they reached out and asked, ‘May I touch it?’ or ‘Can this book be mine?’ We read the book and completed the activities. Everyone applauded the book. Discussions about the book were more frequent compared to regular texts.”

Another observation noted:

“Each page of this week’s book opened up a new story.

-‘Teacher, can I turn the next page?’

-‘Teacher, can I read the next page?’

Students’ requests to read aloud were very important. I allowed them to read even small portions of the text themselves.”

The journal also included one example indicating that students connected the books read during the interactive read-aloud sessions:

*“We read Bears Don’t Read before. Now, we are reading The Book-Loving Bear.*

*In The New Librarian, there was also a bear.”*

According to the qualitative findings from the teacher’s journal, the most liked books were *Library Lion*, *Parsley Rabbit’s Book About Books*, *The Book-Loving Bear*, *Bears Don’t Read*, and *The New Librarian*. The teacher explained the reasons for students’ preference in the journal entry below:

“*After completing all readings, we reviewed the books again. They remembered all the books and events, and overall, they liked the animal-related books the most and were more engaged. Applause is not common in our classroom, but they started clapping themselves during the readings.*”

Another significant point reflected in the teacher’s journal was the development of students’ thinking skills and reading motivation. For instance, the journal notes:

“*Even in a short time, they became excited while reviewing the books again. As the process continued, they began reasoning and participating more actively. Students with reading difficulties also listened and participated. They wanted to touch the books and have them for themselves.*”

## 4. Discussion

The study found that interactive read-aloud activities contributed to the reduction in reading anxiety and the development of positive reading attitudes in second-grade elementary school students. Previous studies had demonstrated that interactive read-aloud activities contributed significantly to literacy and language development through skills such as reading fluency, reading comprehension, reading motivation, engagement in the reading process, and speaking and listening (Ayu et al., 2017; Ceyhan & Yıldız, 2021; Çetinkaya et al., 2018; Martin-Chang & Gould, 2012; Meller et al., 2009; Sezer et al., 2021; Sönmez et al., 2023; Öksüz et al., 2025). The findings of this study make a new contribution to the literature on interactive read-aloud practices within the framework of reading anxiety and attitudes. The qualitative findings further support this contribution by showing that students’ emotional reactions, willingness to participate, and connections with books were consistent with the observed decrease in reading anxiety and increase in reading attitudes.

The decrease in the students’ reading anxiety scores suggests that the interactive read-aloud intervention created a less threatening reading context for second-grade students. Because the students were not required to perform reading individually throughout the entire process, they were able to participate through listening, predicting, discussing, responding to questions, and making personal connections with the texts. This may have reduced the pressure associated with individual oral reading and allowed students to experience reading as a shared and supportive classroom activity. The qualitative findings support this interpretation, as the teacher’s journal indicated that students showed excitement, applauded the books, wanted to touch them, and increasingly volunteered to participate in the reading activities.

The increase in students’ reading attitude scores also indicates that the intervention may have contributed to more positive affective orientations toward reading. The selected picture books focused on books, libraries, reading, and characters who experienced reading-related challenges or enjoyment. Therefore, the content of the books and the interactive nature of the sessions may have helped the students associate reading with enjoyment, curiosity, participation, and social interaction. The teacher’s journal further showed that students remembered the books, expressed preferences for particular stories, and related some book events to their own experiences. These observations suggest that the intervention supported not only measurable changes in reading attitude but also meaningful classroom experiences around books and reading.

Taken together, the quantitative and qualitative findings suggest that interactive read-aloud practices may support the affective dimensions of reading in early primary grades. However, these findings should be interpreted within the limits of the action research design, the single-classroom context, and the absence of a control group. Therefore, the results do not imply that the observed changes can be attributed solely to the intervention; rather, they indicate that interactive read-alouds may be a promising classroom-based practice for reducing reading anxiety and promoting positive reading attitudes among young learners.

## 5. Conclusions

The research findings revealed that interactive read-aloud activities contribute to the development of positive reading attitudes by reducing students’ reading anxiety. Alongside this general finding, it can be inferred that interactive read-aloud interventions can be utilized to foster the affective dimensions of reading. In particular, these practices may serve as a solution in situations where factors such as anxiety act as barriers to reading engagement and can be employed to cultivate positive reading attitudes. This is especially important because even if students are successful in the cognitive aspects of reading, they require support in the affective domain to use reading as a lifelong, sustainable skill.

The integration of interactive read-aloud practices is significant not only for supporting students who experience difficulties in reading but also for enriching the regular second-grade curriculum. Within mainstream classrooms, interactive read-alouds can serve multiple instructional purposes, including vocabulary development, the enhancement of inferential comprehension skills, and the establishment of interdisciplinary connections, while also fostering the affective dimensions of reading. Embedding such sessions as a consistent component of reading instruction has the potential to strengthen curriculum outcomes and promote students’ broader literacy development.

## 6. Limitations

This study has several limitations. First, the study was conducted using an action research design and was tailored to a specific classroom context. This limits the generalizability of the findings to different school types, socioeconomic contexts, or age groups.

The sample consisted of 32 second-grade students attending a public school in Turkey. The relatively small sample size and the use of a single-site sample represent important constraints on the generalizability of the results. In addition, the absence of a control group makes it difficult to attribute the observed changes exclusively to the interactive read-aloud intervention.

The intervention was limited to a 13-week period, which restricts the ability to determine whether the observed effects are sustained over the long term. Similarly, the use of only pre- and post-intervention measurements limits the ability to capture more fine-grained changes throughout the process.

The data collection instruments relied on students’ self-reports. This may pose a limitation in terms of the accuracy and consistency of responses, particularly given the young age of the participants. Furthermore, the qualitative data were based solely on the teacher’s research journal. The use of a single qualitative data source limits the depth and credibility of the process-related findings.

Finally, the fact that the classroom teacher acted both as the practitioner and the data collector may have introduced researcher bias and subjectivity. This should be considered when interpreting the process-oriented findings.

## Figures and Tables

**Table 1:** Bibliographic Information of the Turkish Editions of the Books Used in Interactive Reading Practices.

Book Title	Publisher	Author and Illustrator	Purpose/Theme
Library Lion	Tudem/Uçanbalık Çocuk	Michelle Knudsen Kevin Hawkes	Presents reading as an enjoyable experience and reduces fear or anxiety associated with libraries.
Open This Little Book	MEAV Yayıncılık	Jesse Klausmeier Suzy Lee	Portrays reading as an adventure; encourages enjoyment and relaxation through reading.
The New Librarian	İndigo Çocuk	Alison Donald Alex Willmore	Offers reading as a fun and accessible experience for all children.
Kitabın Yolculuğu (The Journey of the Book)	Yapı Kredi Yayınları	Ezgi Berk Zülal Öztürk	Enhances book appreciation and reading awareness, making reading a meaningful and valuable experience.
Bibliyo Fil (Biblio Elephant)	Timaş Çocuk	V. Hüseyin Kaya	Portrays reading as a fun and safe activity while reinforcing love for books.
Kitap Tamircisi Toprak (Toprak, the Book Repairer)	Final Kültür Sanat Yayınları	Ezgi Berk Ece Zeber	Strengthens love for books and presents reading as a safe and enjoyable activity.
The Highest Mountain of Books in the World	Günışığı Kitaplığı	Rocio Bonilla	Increases curiosity and love for books; presents reading as fun and accessible.
How to Read a Book	Doğan Egmont	Kwame Alexander Melissa Sweet	Makes reading more understandable, enjoyable, and accessible, enhancing reading motivation.
Bears Don’t Read	Arkadaş Yayınevi	Emma Chichester Clark	Presents reading as enjoyable and accessible, promoting love for books.
Madeline Finn and the Library Dog	Hep Kitap	Lisa Papp	Provides a safe and enjoyable reading experience, reduces anxiety, and fosters book appreciation.
Parsley Rabbit’s Book About Books	1001 Çiçek Kitaplar	Frances Watts David Legge	Makes reading enjoyable, educational, and accessible while reinforcing love for books.
The Book-Loving Bear	1001 Çiçek Kitaplar	Eric Pintus Martine Bourre	Offers reading as a fun and accessible experience and strengthens love for books.
Reading Makes You Happy	Beyaz Balina Yayınları	Todd Parr	Presents reading as a joyful and valuable experience, increasing children’s appreciation for books.

**Table 2:** Normality Distribution of the Data.

Variable	N	Mean (M)	Standard Deviation (SD)	Skewness	Kurtosis
Attitude Pre-test	32	3.13	0.419	0.107	−0.867
Attitude Post-test		3.46	0.643	0.220	0.848
Anxiety Pre-test		2.53	1.02	0.932	0.283
Anxiety Post-test		2.04	0.834	0.389	−1.20

**Table 3:** Comparison of Participants’ Pre-test and Post-test Reading Attitude Scores Using a Paired-Samples *t*-Test.

Reading Attitude	*N*	$\bar{x}$	*sd*	*df*	*t*	*p*
Pre-test	32	3.13	0.62	31	−2.98	0.044 *
Post-test	32	3.46				

* *p* < 0.05.

**Table 4:** Comparison of Participants’ Pre-test and Post-test Reading Anxiety Scores Using a Paired-Samples *t*-Test.

Reading Anxiety	*N*	$\bar{x}$	*sd*	*df*	*t*	*p*
Pre-test	32	2.53	0.83	31	3.33	0.002 *
Post-test	32	2.04				

* *p* < 0.05.

**Table 5:** Students’ Responses Observed in the Teacher’s Journal.

Response Type	*f*
Emotional reactions	13
Connecting with personal experiences	9
Willingness to participate in the reading activity	7
Empathizing with the book’s protagonist	3
Focusing on structural features of the book	3
Making intertextual connections	1

## Data Availability

In our current study, in order to protect the confidentiality of participant information and to comply with the confidentiality commitments in the ethics committee approval form (Personal Data Protection Law/informed consent forms), it cannot be shared due to ethical and legal restrictions.
